# Old age is associated with decreased wealth in rural villages in Mtwara, Tanzania: findings from a cross‐sectional survey

**DOI:** 10.1111/tmi.13496

**Published:** 2020-10-18

**Authors:** Salum Mshamu, Pimnara Peerawaranun, Catherine Kahabuka, Jacqueline Deen, Lucy Tusting, Steve W. Lindsay, Jakob Knudsen, Mavuto Mukaka, Lorenz von Seidlein

**Affiliations:** ^1^ CSK Research Solutions Ltd. Dar es Salaam Tanzania; ^2^ Mahidol Oxford Tropical Medicine Research Unit Faculty of Tropical Medicine Mahidol University Bangkok Thailand; ^3^ University of the Philippines Manila Philippines; ^4^ Department of Disease Control London School of Hygiene & Tropical Medicine London UK; ^5^ Department of Biosciences Durham University Durham UK; ^6^ School of Architecture The Royal Danish Academy of Fine Arts Copenhagen Denmark; ^7^ Centre for Tropical Medicine and Global Health Nuffield Department of Medicine University of Oxford UK

**Keywords:** age, wealth accumulation, housing, health, sub‐Saharan Africa

## Abstract

**Objective:**

In many countries, housing is used for wealth accumulation and provides financial security in old age. We tested the hypothesis that household wealth, measured by housing quality and ownership of durable assets, would increase with age of the household head.

**Methods:**

We conducted a survey of household heads in 68 villages surrounding Mtwara town, Tanzania and recorded relevant demographic, housing and social characteristics for each household. The primary analysis assessed the relationship between age of the household head, quality of the house structure and socio‐economic score (SES) using multivariate analysis. Principal Components Analysis (PCA) was used as a data reduction tool to estimate the social‐economic status of subjects based on relevant variables that are considered as proxy for SES.

**Results:**

Of 13 250 household heads were surveyed of whom 49% were male. Those at least 50 years old were more likely to live in homes with an earth floor (86%) compared to younger household heads (80%; *P* < 0.0001), wattle and daub walls (94% *vs*. 90%; *P* < 0.0001) and corrugated iron roofs (56% *vs*. 52%; *P* < 0.0001). Wealth accumulation in the villages included in the study tends to be an inverted V‐relationship with age. Housing quality and SES rose to a peak by 50 years and then rapidly decreased. Households with a large number of members were more likely to have better housing than smaller households.

**Conclusions:**

Housing plays a critical role in wealth accumulation and socio‐economic status of a household in rural villages in Tanzania. Households with a head under 50 years were more likely to live in improved housing and enjoyed a higher SES, than households with older heads. Larger families may provide protection against old age poverty in rural areas. Assuring financial security in old age, specifically robust and appropriate housing would have wide‐ranging benefits.

## Abstract (fr)

**Objectif:**

Dans de nombreux pays, le logement est utilisé pour l'accumulation de richesse et offre une sécurité financière à un âge avancé. Nous avons testé l'hypothèse selon laquelle la richesse des ménages, mesurée par la qualité du logement et la possession d'actifs durables, augmenterait avec l'âge du chef de ménage.

**Méthodes:**

Nous avons mené une enquête auprès des chefs de ménage dans 68 villages entourant la ville de Mtwara, en Tanzanie et enregistré les caractéristiques démographiques, de logement et sociales pertinentes pour chaque ménage. L'analyse primaire a évalué la relation entre l'âge du chef de ménage, la qualité de la structure du logement et le score socioéconomique (SES) à l'aide d'une analyse multivariée. L'analyse en composantes principales (ACP) a été utilisée comme outil de réduction des données pour estimer le statut socioéconomique des sujets sur la base de variables pertinentes qui sont considérées comme une approximation du SSE.

**Résultats:**

13.250 chefs de ménage ont été interrogés, dont 49% de sexe masculin. Les personnes âgées d'au moins 50 ans étaient plus susceptibles de vivre dans des maisons avec un sol en terre (86%) que les chefs de ménage plus jeunes (80%; *P* < 0,0001), des murs en clayonnage enduit de torchis (94% contre 90%; *P* < 0,0001) et des toitures en tôle ondulée (56% contre 52%; *P* < 0,0001). L'accumulation de richesse dans les villages inclus dans l'étude a tendance à être une relation en V inversée avec l'âge. La qualité du logement et le SSE ont atteint un sommet de 50 ans, puis ont rapidement diminué. Les ménages comptant un grand nombre de membres étaient plus susceptibles d'avoir un meilleur logement que les ménages plus petits.

**Conclusions:**

Le logement joue un rôle essentiel dans l'accumulation de richesse et le statut socioéconomique d'un ménage dans les villages ruraux de Tanzanie. Les ménages dont le chef avait moins de 50 ans étaient plus susceptibles de vivre dans un logement amélioré et jouissaient d'un SSE plus élevé que les ménages dont le chef était plus âgé. Les familles plus nombreuses pourraient offrir une protection contre la pauvreté aux personnes âgées dans les zones rurales. Assurer la sécurité financière dans la vieillesse, en particulier un logement solide et approprié, aurait des avantages considérables.

## Introduction

The way households accumulate wealth across nations, social classes and generations are profoundly different. In industrialised countries, wealth generally increases with age and occupation, usually in the form of savings and home ownership, sufficient for the period after retirement [1, 21]. Pension schemes for old age, jointly funded by employers and employees in industrialised countries, are often limited or absent in middle‐ and low‐income countries (MLIC). In many MLIC, family members are expected to provide the financial support and care for the elderly [2]. A critical component of wealth accumulation in middle‐class households is the ownership of real estate. For example, in US households, in 2009, home equity represented 39% of the mean total net worth of the household with other investments such as stocks and mutual funds accounting for 16% [1, 2]. The world’s real estate in 2016 was estimated to be worth USD $228 trillion, more than 60% of all global assets [3].

Land ownership in Tanzania is a mix of public and private as in many other countries, yet the legal situation is complex. According to the Land Act of 1999 [4], land in Tanzania is public land and remains vested in the President as trustee for and on behalf of all citizens of Tanzania. An increasing proportion of urban land is titled, but only 16% of rural land is titled or held with at least some type of formal, written documentation of ownership [5]. In rural areas, a system of communal land rights predominates. The village council headed by the village chairperson, who is elected by the village assembly every five years, distributes user rights among the villagers [6]. Rural households have a strong sense of ownership of their houses, compounds and land even though the legal situation may be more complex. This complexity of communal land rights can make it difficult to obtain mortgages and sell land. Consequently, some household heads try to obtain a title for their land, but the costs for doing so are well beyond the means of most villagers. Even in the absence of titled land and access to mortgages there has been a steady improvement and modernisation of urban and rural housing in Tanzania and, indeed in other parts of sub‐Saharan Africa [7].

The traditional basic rural African house is a single storey structure with an earth floor, walls built of earth and clay, either as mud blocks or plastered onto a stick frame (wattle and daub), and a thatched roof [7]. Variations of this basic structure can be found throughout rural sub‐Saharan Africa. Rural houses with a second storey or elevated on columns, as found throughout rural southeast Asia are rare, and mainly restricted to areas that are water‐logged or subject to flooding. Coincident with the improved economy, villagers modernise their homes in an incremental fashion. Corrugated iron roofs that now dominate most rural landscapes, offer several advantages over thatch, including durability (a thatched roof must be replaced every 3 to 4 years), a reduced fire hazard, and no dust from the decaying thatch falling from the roof. Mud walls must be repaired and plastered over after each rainy season to avoid collapse. Burned bricks and concrete bricks offer longer durability. Concrete floors replace earth floors which can be swept but are rarely clean. In rural Tanzania the prevalence of improved housing with improved water and sanitation, sufficient living area and durable construction has tripled between 2000 and 2015 from 6% to 18% [8].

There is currently little information about the relationship between wealth, as assessed by housing quality, and the age of the household head. Here we tested the hypothesis that the accumulation of wealth during life would follow the model of high‐income countries and result in older household heads having better quality houses than younger household heads. This is highly relevant today since sub‐Saharan Africa has the highest population growth rate in the world [9] and with increasing life expectancy worldwide, there is a growing concern for the welfare of the elderly [10]. In this study, we explored the association between family wealth, as suggested by the quality of housing and ownership of durable assets, and age of the household head in a rural Tanzanian setting.

## Methods

### Study area

The economy of Tanzania is the second‐largest in the East African Community and the tenth‐largest in Africa. Tanzania is largely dependent on agriculture for employment, accounting for about half of the employed workforce. The economy has been transitioning from a state‐directed, command economy to a market economy since 1985 [11]. Although total gross domestic product (GDP) has increased since these reforms began, GDP per capita dropped sharply at first, only exceeded the pre‐transition figure in around 2007, and has been steadily increasing since. After adjusting for purchasing power parity the GDP of Tanzania is $3402 per person per year and ranks globally 154th [12]. About 49% of the population of Tanzania live on less than $1.90/day [13]. The human development index, a composite measure of life expectancy, education and per capita income indicators, is 0.528 and is globally the 159^th^ [14].

The study area is in rural Mtwara (10.3112° S, 40.1760° E), an administrative region of Tanzania that shares its south border with Mozambique. Mtwara region is approximately 16 710 km^2^ in size and in 2012 had a population of 1 270 854 [15]. In Mtwara Region about 90% of the economically active population works in agriculture, mainly cashew nut production. About 70% of cashew nuts produced in the country comes from Mtwara [16]. In 1982, the Italian oil company AGIP discovered the Mnazi Bay gas field in the onshore Rovuma Basin, southern Tanzania [17]. Due to the lack of gas market in Tanzania, the field was not developed until 2006. The hoped for large economic growth from the Mnazi Bay project for Mtwara region has not yet materialised. With a GDP of $2871/person Mtwara region ranks as the 11^th^ highest GDP per capita of 31 regions of Tanzania [18].

### Ethics approval

The studies were approved by the Tanzanian NIMRI ethics review board (Ref. NIMR/HQ/R.8a/Vol. IX/2924). Each respondent provided individual, signed, informed consent; illiterate participants provided a fingerprint countersigned by a literate witness.

### Data collection

Data were collected as part of the baseline assessment of a large housing study (NCT04529434). A team of researchers visited all houses and compounds in 68 villages surrounding Mtwara town. Villages were selected based on the proximity to the town and road access, which is critical for the delivery of building construction materials. The self‐identified household heads were interviewed in the assumption that s/he is most knowledgeable about family wealth and other household issues. The household head provided data his or her own house only. A household was here defined as a person or a group of people, related or unrelated, who live together and share a common source of food. A house is a residence where the family is currently living. The household head is responsible for making decisions within the household, often s/he is also the main provider of the household. A census was conducted in every village, during which time household heads were interviewed, houses were inspected, photographed and geo‐located. A structured form was used to collect demographic and socio‐economic information (Appendix S1). The assets captured by the instrument included personal possessions such as cell phones, watches, housewares such as television set, metal pots, sewing machine, refrigerator, other possessions like bicycles, motorcycles, cars, livestock and immovable assets such as plot for farming. Data were entered in tablets and subsequently uploaded, stored and processed using KoBo Toolbox [19].

### Statistical methods

The primary analysis aimed to assess the relationship between age of the household head, quality of the house structure and socio‐economic score (SES). A Principal Components Analysis (PCA) method was used as a data reduction tool to estimate the social‐economic status of subjects based on relevant variables that are considered as proxy for SES. The housing related variables education level, type of floor, type of walls, type of roof, number of sleeping rooms, type of kitchen, type of toilet, electricity and ownership of durable assets were used in the PCA to estimate the SES. The first principal component was used to construct the model for predicting the socio‐economic score for each respondent as it contains the most information among the PCA factors [21, 22]. The median SES score was then calculated for each age and a median SES was plotted against age. The linear regression analyses were used to draw the fitted lines on median SES score over age for younger and older people. For the break point regression, the optimal R^2^ value was obtained when the cut off was 50 years. A quadratic regression was fitted to confirm the break point which was 49.9 (≈50) years. Respondents under 18 years (n = 19) were excluded from the analysis as they are unlikely to be the true household head and the reliability of responses was, at times, questionable. Poor housing was defined as a house with all of the following criteria an earth floor, mud walls, thatched roof, no electricity and no tap water. For the purposes of this study the ‘younger age group’ was defined as aged 18 to 50 years and the ‘older age group’ from 51 to 98 years, based on visual inspection of the SES/age relationship. A logistic regression model was used to assess the relationship between age group and (younger *vs*. adults) and modern housing defined as a house with concrete/tiles floor, brick walls and metal roof. Significance test was performed at 1% significance level due to multiple comparisons. Analyses were performed in Stata 15.

## Results

**Figure 1:**
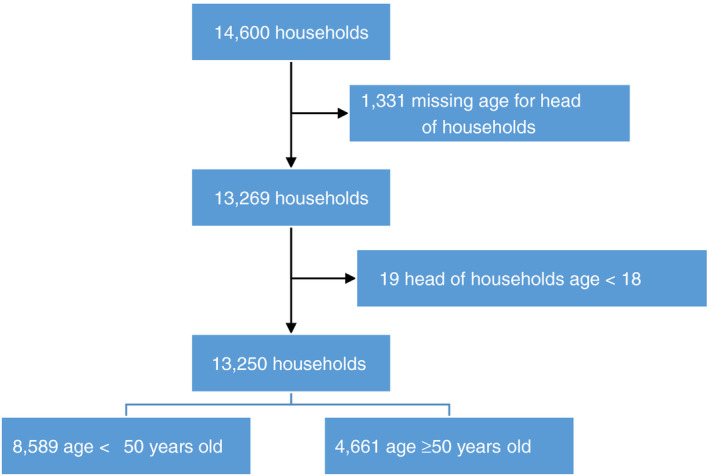
Participant assembly

The study was conducted in 68 villages in Mtwara region, Tanzania between April and August 2019. Of 14 600 villagers approached by study staff 1331 (9%) respondents were excluded because their age was not recorded and 19 (0.1%) were excluded because they were under 18 years (Figure 1). Of 13 250 respondents 4661 (25%) were 50 years or older (Table 1). The younger respondents were more likely to be female (56%) compared to older respondents who were more likely to be male (57%). 46% of the older respondents were illiterate compared to 31% of the younger respondents (*P* < 0.0001). 68% of the older respondents were married which is lower compared to 75% of the younger respondents (*P* < 0.0001). And almost all (94%) of the older respondents were farmers which is higher compared to 86%, younger respondents (*P* < 0.0001). 41% and 32% of the households had 3 or 4 rooms, respectively. Those that had only 2 rooms accounted for 20% of the total.

**Table 1:** Characteristics of respondents (n = 13 250)

Characteristics	Age < 50 years old	Age ≥ 50 years old	Total	*P*‐value
n	8589	4661	13 250	
n (%)				
Sex				<0.0001
Female	4769 (56)	2026 (43)	6795 (51)	
Male	3820 (44)	2635 (57)	6455 (49)	
Education				<0.0001
Illiterate	2684 (31)	2147 (46)	4831 (36)	
Literate, no formal education	417 (5)	308 (7)	725 (5)	
Primary school	4900 (57)	2121 (46)	7021 (53)	
Secondary school	534 (6)	66 (1)	600 (5)	
High School/Colleges/Vocation training/Graduate (After High School)	54 (1)	19 (0)	73 (1)	
Marital status				<0.0001
Married	6427 (75)	3162 (68)	9589 (72)	
Cohabiting	141 (2)	47 (1)	188 (1)	
Separated	524 (6)	349 (7)	873 (7)	
Divorced	397 (5)	248 (5)	645 (5)	
Widow/Widower	160 (2)	559 (12)	719 (5)	
Single	940 (11)	296 (6)	1236 (9)	
Occupation				<0.0001
Farmer	7367 (86)	4396 (94)	11 763 (89)	
Fisherman	357 (4)	68 (1)	425 (3)	
Trader/Selling goods	423 (5)	39 (1)	462 (3)	
Employee	79 (1)	26 (1)	105 (1)	
Craft, Clothes or Furniture	97 (1)	8 (0)	105 (1)	
Labourer	48 (1)	7 (0)	55 (0)	
Others	105 (1)	109 (2)	214 (2)	
Don’t know	113 (1)	8 (0)	121 (1)	

Older household heads were more likely to live in homes with an earth floor (86%) compared to younger household heads (80%; *P* < 0.0001), and wattle and daub constructions (94% *vs*. 90%; *P* < 0.0001). The frequency of thatched roofs was lower among the older than younger respondents (42% *vs*. 46%; *P* < 0.0001; Table 2). The large majority of homes was owned by the household heads and their families overall, only 4% of homes were rented. Approximately 55% of the older respondents used outdoor kitchens compared to 48% of the younger respondents (*P* < 0.0001). The most frequently used type of toilet (85%) was a pit latrine with an earth floor irrespective of the age of the household head. The three most common water sources were a community well (43%), water collected from a lake or river (16%), or a public tab (11%). 49% of the younger respondents had access to electricity but only 42% of the respondents at least 50 years. Older respondents were less likely to own durable assets like cell phones (63% *vs*. 75%; *P* < 0.0001); television (4% *vs*. 9%; *P* < 0.0001), motorbikes (7% *vs*. 10%; *P* < 0.0001) and bicycles (41% *vs*. 43%; *P* = 0.011).

**Table 2:** The quality of housing, water, sanitation and durable assets according to the respondents (n = 13 250)

Characteristics	Age < 50 years old	Age ≥ 50 years old	Total	*P*‐value
n	8589	4661	13 250	
	n (%)			
**Quality housing**				
Type of floor				<0.0001
Concrete/Cement/Tiles	1539 (18)	590 (13)	2129 (16)	
Earth	6909 (80)	4028 (86)	10 937 (83)	
Other	141 (2)	43 (1)	184 (1)	
Type of walls				<0.0001
Burned Clay Bricks	193 (2)	39 (1)	232 (2)	
Concrete Blocks	332 (4)	113 (2)	445 (3)	
Mud and Bricks	233 (3)	72 (2)	305 (2)	
Wattle and daub	7706 (90)	4373 (94)	12 079 (91)	
Others	125 (1)	64 (1)	189 (1)	
Type of roof				<0.0001
Corrugated Iron	4504 (52)	2632 (56)	7136 (54)	
Thatched roof	3950 (46)	1974 (42)	5924 (45)	
Others	135 (2)	55 (1)	190 (1)	
**Ownership of home**				<0.0001
Owner	6858 (80)	4107 (88)	10 965 (83)	
Family owned	583 (7)	196 (4)	779 (6)	
Owned by a relative	515 (6)	172 (4)	687 (5)	
Rented	481 (6)	111 (2)	592 (4)	
Others	152 (2)	75 (2)	227 (2)	
**Number of rooms for sleeping**				<0.0001
0–1	1400 (16)	716 (15)	2116 (16)	
2	4083 (48)	1895 (41)	5978 (45)	
3 or more	3106 (36)	2050 (44)	5156 (39)	
**Type of kitchen**				<0.0001
Indoor kitchen	4442 (52)	2108 (45)	6550 (49)	
Outside kitchen	4147 (48)	2553 (55)	6700 (51)	
Poor housing (earth floor, wattle & daub/mud wall, thatched roof, no electricity, no tab water)	2130 (25)	1301 (28)	3431 (26)	<0.0001
**Water supply and sanitation**				
Type of toilet				0.002
Flush toilet inside the house	34 (0.4)	11 (0.2)	45 (0.3)	
Pit latrine with cement floor	328 (4)	131 (3)	459 (4)	
Pit Latrine with earth floor	7259 (85)	4046 (87)	11 305 (85)	
Communal pit latrine	321 (4)	162 (4)	483 (4)	
The bush/open defecation	386 (5)	202 (4)	588 (4)	
Others	261 (3)	109 (2)	370 (3)	
**Main water source**				0.032
Piped into the house	9 (0.1)	4 (0.1)	13 (0.1)	
Public tap	953 (11)	478 (10)	1431 (11)	
Individual shallow well	212 (3)	136 (3)	348 (3)	
Purchased	401 (5)	213 (5)	614 (5)	
Bore hole	107 (1)	78 (2)	185 (1)	
Community well	3784 (44)	1959 (42)	5743 (43)	
Collected from a river/lake	1297 (15)	753 (16)	2050 (16)	
Rainwater collection	315 (3.7)	200 (4.3)	515 (3.9)	
Others	1511 (18)	840 (18)	2351 (18)	
**Electricity** (access to grid)				<0.0001
No	4374 (51)	2688 (58)	7062 (53)	
Yes	4215 (49)	1973 (42)	6188 (47)	
**Ownership of durable assets**				
Cell phone	6439 (75)	2951 (63)	9390 (71)	<0.0001
Radio	3364 (39)	1808 (39)	5172 (39)	0.671
Television	738 (9)	207 (4)	945 (7)	<0.0001
Motorbike	885 (10)	322 (7)	1207 (9)	<0.0001
Bicycle	3726 (43)	1915 (41)	5641 (43)	0.011
**Ownership land and livestock**				
Plot acres*	2 (1, 3)	2.5 (1, 4)	2 (1, 3)	<0.0001
Livestock (goat/cows/chicken)	4517 (53)	2536 (54)	7053 (53)	0.045

a Is median (IQR), but the rest are n (%).^*^

**Figure 2:**
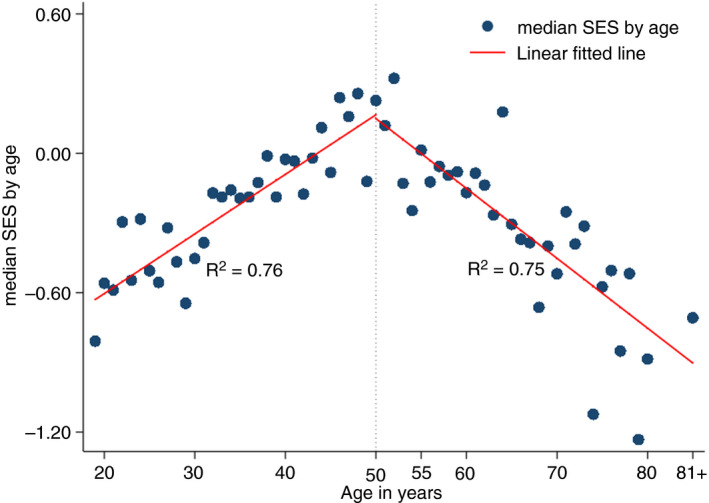
Age of household head and socio‐economic status (SES) of the household

In the PCA, the three most important components for a high socio‐economic score were related to the quality of housing namely roofing materials, location of the kitchen, floor materials (Table 3). Other relevant components included access to electricity, ownership of a television, number of bed rooms, motorbikes, cell phones, brick walls, plot size, flush or pit latrine with cement floor, bicycle, livestock and literacy. The median SES score increased from 18 years reached an apex around 50 years and then declined. The linear regression analyses showed that there was positive relationship between SES and age in younger household heads (*r* = 0.87) and there was negative relationship between SES and age in older household heads (*r* = −0.87) (Figure 2). Households with younger household heads were more likely to have improved housing OR 2.0, (95% CI 1.6, 2.6) compared to older household heads.

**Table 3:** Principal component analysis (N = 13 250)

Factors	Factor score
Corrugated Iron Roof	0.363
Outside Kitchen	0.338
Concrete/Cement/Tiles Floor	0.321
Electricity	0.314
TV	0.278
Having 3 or more Sleeping rooms	0.262
Motorbikes	0.261
Cell phones	0.240
Radios	0.228
Backed Bricks/Concrete blocks Walls	0.212
Having 3 or more Plot acres	0.209
Flush/ pit latrine with cement floor Toilet	0.208
Bicycle	0.205
Livestock (goats/cows/chicken)	0.183
Illiterate status	0.156

**Figure 3:**
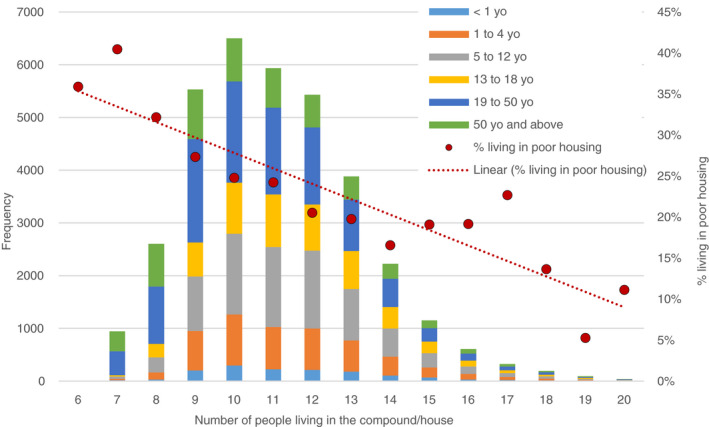
Number of residents, their age group and the quality of housing (poor housing) (*y* = −0.0188*x* + 0.3723; *R* ^2^ = 0.8266)

The respondents reported that their households included between 6 and over 20 members. The age distributions of the reported household members as illustrated in Figure 3 showed an inverse correlation between the number of reported household members living in the entire household and the proportion of people living in poor housing; the larger the number of household members the less likely that their house had a poor structure.

## Discussion

We found that wealth accumulation in the study villages peaks and levels off by age 50 years, after which it decreases precipitously. Households with a head under 50 years were more likely to live in improved housing and enjoyed a higher socio‐economic status. Households with a large number of members were more likely to have better housing compared to smaller households. These observations suggest that the poverty‐related problems faced by residents of the area are most severe among the elderly living on their own.

**Figure 4:**
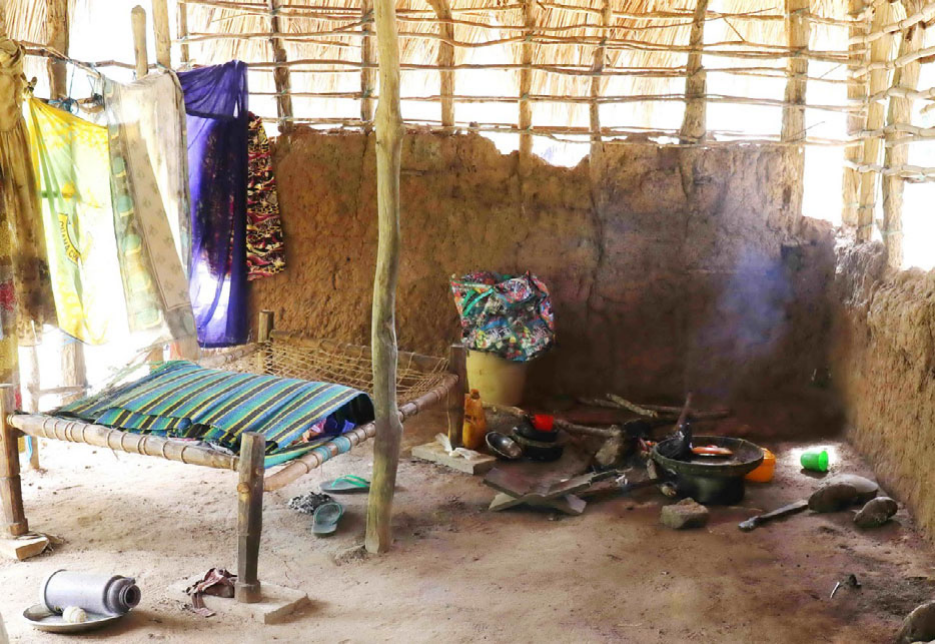
The collapsing home of an elderly woman in Mtwara region, Tanzania

Most people in the study villages work in agriculture. The nature of work is highly demanding and takes its toll on the health of the workers. By the time they are 60 years most people find it hard to continue demanding physical work. Households that depend in large part on the income of the household head experience a decrease in income once the head reaches such an age. Maintaining a basic wattle and daub home is labour‐intensive. An earth floor needs to be swept several times daily, mud walls need to be repaired at least after each rainy season, otherwise the walls collapse (Figure 4). Thatched roofs need to be replaced every three to four years, otherwise they provide little protection from rainfall which in turn accelerates the deterioration of mud walls. Older people left on their own are often unable to complete these physically demanding repairs. In the absence of money to pay workers, the home deteriorates rapidly and collapses (Figure 4). Large households that include younger adults provide some protection against age‐related poverty as they can contribute financially, repair structural problems in the home and eventually can help replace the home once repairs are no longer feasible. Our observations suggest that support from neighbours and fellow villagers does not assure protection against poverty in old age.

Our study has several limitations. First, as mentioned earlier the economy of Tanzania has undergone profound changes over the last 40 years including a transition from command to market economy. This leaves the theoretical possibility that people reaching adulthood during this transition remained economically disenfranchised while the following generation benefitted from the opening of economic opportunities. This scenario suggests that wealth or more precisely the relative poverty of the people over 50 is fixed. Our study design, a survey at a single point in time cannot distinguish whether wealth in study sites is fixed or dynamic. But based on the experience of the investigators it seems more likely that wealth is dynamic and directly correlates with the income capacity from labour which declines with age. Second while the housing structures can be directly observed for other information, we have to rely on the respondents’ information. This may explain the surprisingly large number of reported household members. As the respondents were asked about the members belonging to the household family members who have left the villages long ago and show no inclination to return any time soon were still included. The reported number of household members has to be understood as the ideal household as imagined in the mind of the respondent and not the *de facto* household. Third, not all assets could be checked, and it is possible that land ownership and ownership of durable assets are overstated. We believe these limitations are outweighed by the strengths of the study, which includes its reliance on original primary data and the reporting of the house structure by direct observation.

The findings of this study provide an explanation for the preference of large households and big families which have resulted in the rapid population expansion in many parts of Africa. It seems likely that in many rural African villages the only protection against old age poverty is the hope that younger household members will eventually take care of the household. We are currently exploring new housing constructions for rural Africa that aside from providing a healthy environment, will be more durable compared with the current local standard. It is likely that a long‐lasting structure may contribute towards protection against old age poverty by assuring a safe refuge for the ageing members of a household.

## Supporting information


**Appendix S1** SHT2 ELIGIBILITY SURVEY – structured questionnaire.

## Data Availability

Data are available on request from CSK Research Solutions Ltd., Dar es Salaam, Tanzania; ckahabuka@cskresearch.com.
