# Supplementary material for: Old age is associated with decreased wealth in rural villages in Mtwara, Tanzania: findings from a cross‐sectional survey
Source: Trop Med Int Health. 2020 Oct 18;25(12):1441–9. doi: 10.1111/tmi.13496 (PMC7756872; doi:10.1111/tmi.13496)
Supplement: Supplementary file 1 — Appendix S1 SHT2 ELIGIBILITY SURVEY – structured questionnaire. [file TMI-25-1441-s001.docx]

# SHT2 ELIGIBILITY SURVEY – structured questionnaire

# Part **A:** Demographics

1. Respondent ID number/Household number: ________________________________
2. Head of household name: _________________________________
3. Date of Interview *(dd/mm/yy*) __________________
4. Interviewer code: ________________________________
5. Village Name: ____________________________
6. Household location___________________________
7. Is the respondent the head of the household? *1. Yes 2. No*
8. Respondent relationship to the head of the household 1. Wife 2. Husband 3. Child 4. Grandparent 5. Other; mention ___________________
9. Respondent Age in years__________ *(write age)*
10. Respondent Gender: *1. Female 2. Male*
11. Marital Status: *1. Married/Cohabiting 2. Single 3. Divorced/Separated 4. Widow/Widower*
12. Religion: *1. Muslim 2. Christian 3. Traditional 4. No religion 5. No response 6. Other ___________________*
13. Level of education of the head of the household?

*1. Illiterate*

2. *Literate, no formal education*

3*. Primary School*

4. *Secondary School*

5. *Colleges / Vocational trainings*

6. *High school*

7. *Graduate (after high school)*

1. What is the occupation of the head of the household?
2. Fisherman
3. Trader / Selling goods
4. Farmer
5. Service worker
6. Labourer
7. Craft, clothes, or furniture maker
8. Technician
9. Other: Mention

PART B: ASSESSMENT FOR INCLUSION

*Family:*

1. Who lives in this household (include all who live here 6 months a year or more)? *(Write the number of household members within each age category)*

____ Infants under 1 year

____ Children 1 to 4 years

____ Children 5 to 12 years

____ Adolescents 13 to 18 years

____ Adults 19 to 50 years

____ Adults 51 and older

1. How many children under 12 years are currently traveling or studying in other places but whom you expect to return to the village?
2. For how many years have you been living in this village? Years [____________]
3. For how long are you planning to be living in this village?

*1. Less than 3 years*

*2. More than 3 years*

1. Are you willing to participate in the health survey which will last for 3 years after you have moved into the new house? (This will mean research assistants visiting your house once a week for 3 years)
2. Yes 2. No

*Housing and facilities (Observe and record)*

1. What are the primary construction materials used in the respondents’ current house?

**Floor:** *1. Earth 2. Concrete 3. Tiles 4. Other: mention ______________*

**Walls:** *1. Mud & wooden sticks 2. Mud bricks 3. Baked bricks 4. Concrete blocks 5. Other; Mention* ____________

**Roof:** *1. Thatched roof 2. Corrugated iron sheets 3. Concrete 4. Mud roof 5. Roof tiles 6. Other: mention*______________

1. Ownership of housing? *1. Rented 2. Owner 3. Relative owned 4. Family owned 5. Other: mention _____________*
2. How many rooms are in the house? [________]
3. How many rooms are used for sleeping? [­­­­­_______]
4. What type of kitchen do you have? (Observe the position of the cooking area)

*1. Inside kitchen (Main house)*

*2. Outside kitchen (next to the main house)*

1. Does your household own a toilet? 1. Yes 2. No
2. If yes, what kind of toilet do the adult household members usually use?
3. Flush toilet inside the house
4. Communal flush toilet
5. Communal pit latrine with cement floor
6. Communal pit latrine with earth floor
7. Pit Latrine with earth floor – of single household
8. Pit latrine with cement floor – of single household
9. The bush
10. Anywhere
11. Other: mention ___________
12. What is the main source of water in the house?
13. Piped into the house
14. Stand pipe outside the house
15. Stand pipe - public tap
16. Bore hole
17. Individual shallow well
18. Community shallow well
19. Rain water collection
20. Standing water from the rain
21. Purchased
22. Collected directly from a lake/river
23. Other: Mention ________________
24. Does your house have electricity? 1. Yes 2. No
25. If “Yes” what is the source of electricity?
26. Solar 2. Grid (Tanesco) 3. Other; mention ______________
27. Do you anticipate that your house may get electricity within the next two years? 1. Yes 2. No
28. Do you have an area next to your house where you can construct a house? (Observe the construction plot) 1. Yes 2. No

PART C: SOCIO-ECONOMIC ASSESSMENT

1. Does your household own any of the following? [Record the quantity in the provided space]
2. Cellular phone  1. Yes; How many? [______] 2. No
3. Working watches/clocks 1. Yes; How many? [______] 2. No
4. Radio 1. Yes; How many? [______] 2. No
5. Plot for planting 1. Yes; How big (Acres)? [_ ] 2. No
6. Refrigerator 1. Yes; How many? [______] 2. No
7. Sewing machine 1. Yes; How many? [______] 2. No
8. Metal pots 1. Yes; How many? [______] 2. No
9. Goats 1. Yes; How many? [______] 2. No
10. Television 1. Yes; How many? [______] 2. No
11. Car/vehicle 1. Yes; How many? [______] 2. No
12. Mosquito nets 1. Yes; How many? [______] 2. No
13. Cows 1. Yes; How many? [______] 2. No
14. Motorbike 1. Yes; How many? [______] 2. No
15. Bicycle 1. Yes; How many? [______] 2. No
16. Solar power 1. Yes; How many? [______] 2. No
17. Chicken 1. Yes; How many? [______] 2. No
